# Synthesis of Polystyrene-Coated Superparamagnetic and Ferromagnetic Cobalt Nanoparticles

**DOI:** 10.3390/polym10101053

**Published:** 2018-09-20

**Authors:** Li Tan, Bing Liu, Konrad Siemensmeyer, Ulrich Glebe, Alexander Böker

**Affiliations:** 1 Fraunhofer Institute for Applied Polymer Research IAP, Geiselbergstr. 69, 14476 Potsdam-Golm, Germany; li.tan@iap-extern.fraunhofer.de; 2 Lehrstuhl für Polymermaterialien und Polymertechnologie, Universität Potsdam, 14476 Potsdam-Golm, Germany; 3 Institute of Chemistry Chinese Academy of Sciences, Beijing 100864, China; liubing@iccas.ac.cn; 4 Helmholtz-Zentrum Berlin, Hahn-Meitner-Platz 1, 14109 Berlin, Germany; siemensmeyer@helmholtz-berlin.de

**Keywords:** cobalt nanoparticles, polystyrene, superparamagnetic, ferromagnetic, molecular weight

## Abstract

Polystyrene-coated cobalt nanoparticles (NPs) were synthesized through a dual-stage thermolysis of cobalt carbonyl (Co_2_(CO)_8_). The amine end-functionalized polystyrene surfactants with varying molecular weight were prepared via atom-transfer radical polymerization technique. By changing the concentration of these polymeric surfactants, Co NPs with different size, size distribution, and magnetic properties were obtained. Transmission electron microscopy characterization showed that the size of Co NPs stabilized with lower molecular weight polystyrene surfactants (*M*_n_ = 2300 g/mol) varied from 12–22 nm, while the size of Co NPs coated with polystyrene of middle (*M*_n_ = 4500 g/mol) and higher molecular weight (*M*_n_ = 10,500 g/mol) showed little change around 20 nm. Magnetic measurements revealed that the small cobalt particles were superparamagnetic, while larger particles were ferromagnetic and self-assembled into 1-D chain structures. Thermogravimetric analysis revealed that the grafting density of polystyrene with lower molecular weight is high. To the best of our knowledge, this is the first study to obtain both superparamagnetic and ferromagnetic Co NPs by changing the molecular weight and concentration of polystyrene through the dual-stage decomposition method.

## 1. Introduction

Due to a variety of applications in biological imaging, therapeutic applications and magnetic energy storage [1,2,3,4], the preparation of magnetic nanoparticles (NPs) has been widely investigated. Decomposition of metallic precursors at high temperature is a general method to synthesize well-defined magnetic nanoparticles. Up to now, numerous studies about the synthesis of magnetic nanoparticles with small molecules (e.g., oleic acid, oleylamine, trioctylphosphine oxide) as stabilizer have been reported [5,6,7,8,9,10]. The size and shape of the nanoparticles can be influenced by many factors (e.g., temperature, reaction time, and the concentration of the precursor or stabilizer) [5,7,9,11,12,13,14]. In addition to small molecule stabilizer, magnetic nanoparticles with polymeric stabilizer have also been prepared [15,16,17,18,19,20]. On one hand, the polymeric stabilizers provide advantages over small molecules due to the stronger steric repulsive force to stabilize magnetic nanoparticles [21]. On the other hand, the composition of the polymer can be tuned through copolymerization or post-functionalization [22]. Thus, the approach becomes more attractive due to the diversity of polymer properties, which enable magnetic nanoparticles with a variety of functions and applications in many fields, such as drug delivery, data storage, and medical applications [23,24,25]. Furthermore, the development of controlled/living free radical polymerization enables the synthesis of a wide range of polymers with precise molecular weight and composition, which exhibit prominent advantages for the synthesis of magnetic nanoparticles/shell hybrids with tunable thickness and composition [26,27]. For example, Smith et al. reported that magnetic iron nanoparticles stabilized with vinyl polymers were prepared through a thermolysis of Fe(CO)_5_ and showed that polymer molecules can be used as an effective stabilizer for the dispersion of NPs [20]. However, due to the rapid oxidation of iron nanoparticles, which led to a great reduction of the magnetic moment, more attention was paid to the preparation of iron oxide and cobalt nanoparticles [15,16,17,19]. The cobalt bulk material has a higher saturation magnetization (161 emu/g) than that of magnetite (Fe_3_O_4_) (92 emu/g) [28], and thus it usually leads to Co NPs with a better magnetic response for many applications. 

The size of magnetic nanoparticles plays an important role for their magnetic properties. Generally, ferromagnetic NPs are larger than superparamagnetic NPs. The magnetic properties of ferromagnetic NPs are dominated by a hysteresis loop exhibiting remanence and coercivity. Ferromagnetic NPs usually self-assemble into one-dimensional (1-D) chain structures due to dipolar interaction [15,29]. When the size of the NPs is reduced below a critical value, the particles become superparamagnetic [30], exhibiting no residual magnetization after removal of the external magnetic field. Therefore, superparamagnetic NPs do not agglomerate without external magnetic field which is a prerequisite for some medical applications. Thus, the control of the size of magnetic NPs is essential. In the preparation of magnetic NPs through the thermal decomposition of metal precursors, two steps are involved in the process: the formation of nucleation nanocrystals by decomposition of the metal precursor at high temperature, followed by the growth of the nuclei. Therefore, many parameters can affect the size and morphology of magnetic NPs, including temperature, surfactants (composition, molecular weight, and concentration), reaction time, solvent, etc. Up to now, some efforts have been made to control the size and morphology of Co NPs [31,32,33]. For example, Pyun et al. synthesized magnetic Co NPs by using different end-functional polystyrene surfactants through thermal decomposition of cobalt carbonyl (Co_2_(CO)_8_) [31,32,33]. By varying the reaction temperature, ferromagnetic Co NPs with different sizes ranging from 18 to 43 nm were obtained [33]. Different morphologies of Co NPs were observed when using different molecular weight of polystyrene [31]. However, the preparation of superparamagnetic Co NPs by using polystyrene surfactant has rarely been reported. Herein, we show that both superparamagnetic and ferromagnetic Co NPs can be synthesized by employing end-functionalized polystyrene as stabilizer via a dual-stage thermolysis of Co_2_(CO)_8_. In addition, we studied the influence of the polymers’ molecular weight on the grafting density of the hybrid particles in detail. 1-D assemblies of such ferromagnetic hybrid nanoparticles can be applied in bottom-up materials synthesis and as building blocks for mesoscopic assemblies [31], while the superparamagnetic Co NPs can be used as ferrofluid.

## 2. Materials and Methods 

### 2.1. Materials

*N*-hexane (95%), THF (99.9%), and dichloromethane were purchased from Th. Geyer (Renningen, Germany); methanol and acetonitrile (HPLC-grade) were purchased from VWR (Darmstadt, Germany)); *N*,*N*-dimethylformamide (DMF, 99%) was purchased from Acros Organics (Geel, Belgium); anhydrous 1,2-dichlorobenzene (DCB, 99%), methanol (HPLC-grade), cobalt carbonyl (Co_2_(CO)_8_) containing 1%–5% *n*-hexane as stabilizer, hydrazine monohydrate (N_2_H_2_ 64%–65%), 2,2′-bipyridyl (99%), styrene (99%), α,α′-dichloro-*p*-xylene (98%), 18-crown-16 (99%), phthalimide potassium salt (99%), copper(I) chloride (97%), and alumina (activated, neutral, Brockmann Activity I) were purchased from Sigma Aldrich (Steinheim, Germany) and used as received without further purification. Copper(I) chloride was purified by stirring in glacial acetic acid overnight, washed with ethanol and dried under vacuum. Co_2_(CO)_8_ was kept in the fridge of a glovebox, and *n*-hexane evaporated in the glovebox before use. Styrene was passed through a short column of neutral alumina to remove inhibitors and deoxygenated by bubbling with N_2_ before starting a polymerization. 

### 2.2. Synthesis of Amine End-Functionalized Polystyrene (PS-NH_2_)

#### 2.2.1. Synthesis of 4-(Chloromethyl)benzyl Phthalimide 2

The synthesis of compound 2 was performed according to the literature [32]. In detail, in a 150 mL round-bottom flask, α,α′-dichloro-*p*-xylene (6.59 g, 376 mmol), phthalimide potassium salt (2.67 g, 144 mmol) and 18-crown-6 (380 mg, 1.43 mmol) were dissolved in 75 mL acetonitrile. The reaction was refluxed overnight under the protection of N_2_. After the reaction mixture cooled down to room temperature, the solvent was evaporated under vacuum. The resulting crude product was purified by column chromatography on silica gel (CH_2_Cl_2_:hexane = 5:1), giving a colorless crystalline solid **2** (2.0 g, 49% yield). ^1^H-NMR (400 MHz, CDCl_3_): δ = 7.85 (m, Ar–H, 2H), 7.71 (m, Ar–H, 2H), 7.45–7.40 (m, Ar–H, 2H), 7.36–7.31 (m, Ar–H, 2H), 4.84 (s, CH_2_, 2H), 4.54 (s, CH_2_, 2H).

#### 2.2.2. Synthesis of Benzyl Phthalimide End-Functionalized Polystyrene **3**

In a 100 mL Schlenk flask, compound **2** (500 mg, 1.75 mmol), 2,2′-bipyridyl (546 mg, 3.5 mmol), and CuCl (173 mg, 1.75 mmol) were added. The flask was sealed with a rubber septum, evacuated and back-filled with N_2_ for three cycles. 2 mL degassed DMF was injected, followed by the addition of degassed styrene (24 mL, 210 mmol). The flask was placed into an oil bath at 110 °C for 16 h. After cooling down to room temperature, the flask was opened to air. The reaction mixture was diluted with 300 mL dichloromethane, and passed through a neutral alumina plug to remove the copper catalyst. The collected solution was concentrated under vacuum and precipitated into 1 L methanol twice. The resulting colorless powder was dried in vacuum. ^1^H-NMR (400 MHz, CDCl_3_): δ = 7.82 (b, Ar–H, 2H), 7.68 (b, Ar–H, 2H), 7.2–6.3 (m, Ar–H), 4.77 (m, CH_2_, 2H), 2.4–1.2 (m, CH + CH_2_). *M*_n,GPC_ = 4500 g/mol; *M*_w_/*M*_n_ = 1.28.

To synthesize benzyl phthalimide end-functionalized polystyrene of different molecular weight, the procedure was conducted the same, only by changing the amount of monomer styrene and the reaction time. Twelve mL styrene (105 mmol) and 14 h reaction time resulted in a polymer of *M*_n_ = 2300 g/mol; *M*_w_/*M*_n_ = 1.38. Similarly, 24 mL styrene (105 mmol) and 41 h resulted in a polymer of *M*_n_ = 10,500 g/mol; *M*_w_/*M*_n_ = 1.17.

#### 2.2.3. Synthesis of Amine End-Functionalized Polystyrene **4** (PS-NH_2_)

In a 100 mL round-bottom flask, benzyl phthalimide end-functionalized polystyrene (1.8 g, 0.4 mmol) was dissolved in 30 mL THF. Three mL methanol was added dropwise, and hydrazine monohydrate (640 mg, 12.8 mmol) was slowly dropped. The reaction mixture was allowed to stir at room temperature overnight until a precipitate was observed. The reaction mixture was concentrated and redissolved in dichloromethane, followed by the precipitation in 1 L methanol twice. The resulting white powder was dried under vacuum. ^1^H-NMR (400 MHz, CDCl_3_): δ = 8.5 (s, NH_2_, 2H), 7.2–6.3 (m, Ar–H), 3.9 (m, CH_2_, 2H), 2.4–1.1 (m, CH + CH_2_).

The other amine end-functionalized polystyrenes with different molecular weight were prepared by the same procedure.

### 2.3. Synthesis of PS-Co NPs with Amine End-Functionalized Polystyrene as Surfactant

For the synthesis of PS–Co NPs, PS–NH_2_ of different molecular weight and concentration was used. Typically, in a clean and dried 100 mL three-neck round bottom flask, PS-NH_2_ (*M*_n_ = 4500 g/mol, 225 mg, 0.5 mmol) was added. The flask was then equipped with a condenser, and placed in an oil bath. The whole system was evacuated and back-filled with N_2_ three cycles. Degassed 1,2-dichlorobenzene (18 mL) was injected, and the solution was heated to 175 °C. One hundred and fifty mg Co_2_(CO)_8_ in 2 mL of DCB was rapidly injected at the reflux temperature. After 10 min, the temperature was lowered to 160 °C and the solution was stirred for another 30 min. The reaction mixture was cooled to room temperature under N_2_ atmosphere, then, the suspension was collected with a syringe, and transferred into a Schlenk tube which was deoxygenated beforehand and stored in the fridge. To purify the nanoparticles, they were precipitated in *n*-hexane and centrifuged to remove the supernatant. The average particle size was determined by TEM through ImageJ software (1.52 b, National Institute of Health, Bethesda, MD, USA). 

### 2.4. Instruments

Transmission electron microscopy (TEM) images and high resolution TEM images were obtained by using a Titan 80-300 microscope (FEI, Hillsboro, OR, USA) operated at 300 kV. The samples were prepared by drop-casting a NP suspension onto the carbon-coated copper grid, followed by drying in air. The size of the Co NPs was estimated on TEM images using ImageJ software fitting the particles with ellipsoid shapes and the average size and standard deviation of each sample were obtained from a sample of around 200 NPs. Thermogravimetric analyses (TGA) were conducted on a TA Instruments Q500 (TA Instruments, Eschborn, Germany). All samples were measured from 25 to 800 °C under a nitrogen atmosphere with a heating rate of 10 °C/min. Wide angle X-ray scattering (WAXS) measurements were done by means of a Bruker-AXS texture goniometer (D5000 equipped with a closed Eulerian Cradle) in reflection mode. Ni-filtered Cu-Kα radiation operated at 30 mA and 40 kV was applied. Scattering intensities were recorded in the 2θ-range from 40° to 55° with a scan speed of 55 s/step and with a step size Δθ of 0.015° (total measurement time: 15 h 17 min). The samples were prepared as powder samples (manually in a mortar) and then put in an appropriate sample holder. The magnetic properties of Co NPs were measured using a SQUID magnetometer (MPMS 3, by Quantum Design) at the Quantum Materials Core Lab at the Helmholtz Zentrum Berlin, Germany. The magnetic behavior of Co NPs was measured in field cooling (FC) and zero-field cooling (ZFC) modes from 5 to 390 K under an applied field of 50 mTesla. Magnetization curves were obtained under the fields from 0–2 Tesla. The magnetic data were normalized from the sample mass of Co atoms. Polymer number-average molecular weight (*M*_n_, GPC), weight-average molecular weight (*M*_w_, GPC), and molecular weight distribution (also polydispersity index, PDI) were determined by gel permeation chromatography (GPC) using DMF with LiBr (1 mg/mL) as eluent, a flow rate of 1.0 mL/min, a high pressure liquid chromatography pump (Spectra Systems P 1000), and a refractometer SEC-3010 (WGE Dr. Bures) detector. Calibration was achieved using polystyrene standards. Results were evaluated using Parsec 5.62 (Brookhaven Instruments) software (Lang Island, New York, USA). Matrix-assisted laser desorption/ionization time-of-flight (MALDI-ToF) mass spectra were collected on a 337 nm Bruker microflex spectrometer (Bruker, Bremen, Germany) with pulsed ion extraction. The masses were determined in positive ion linear mode. The sample solutions were applied on a ground steel target using the dried droplet technique. THF was used as solvent for PS (5 mg/mL), matrix dithranol (10 mg/mL), and sodium trifluoroacetate (13.6 mg/mL). Sample, matrix, and salt solutions were mixed in 5:20:1 ratio and 2 µL of the mixture applied on the target. Proton nuclear magnetic resonance (^1^H-NMR) spectra were measured on a Bruker DPX-400 spectrometer at 400 MHz. All samples were prepared in deuterated chloroform (CDCl_3_, 99.8%). All spectra were referenced to the signal of non-deuterated solvent relative to tetramethylsilane = 0 ppm.

## 3. Results and Discussion

### 3.1. Synthesis and Characterization of PS-NH_2_

**Figure 1:**
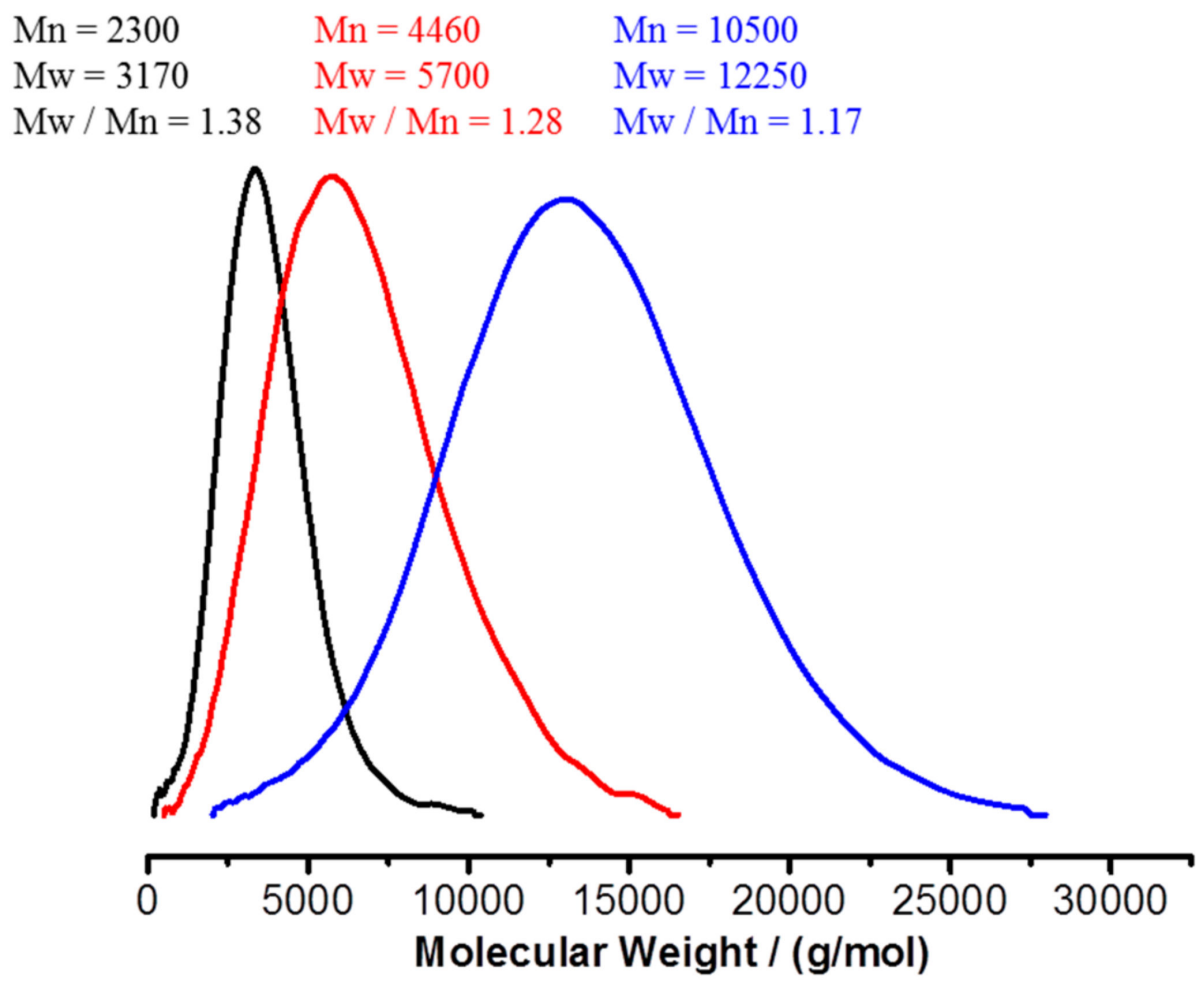
Gel permeation chromatography (GPC) curves of PS–NH_2_ with different molecular weight.

**Scheme 1:**
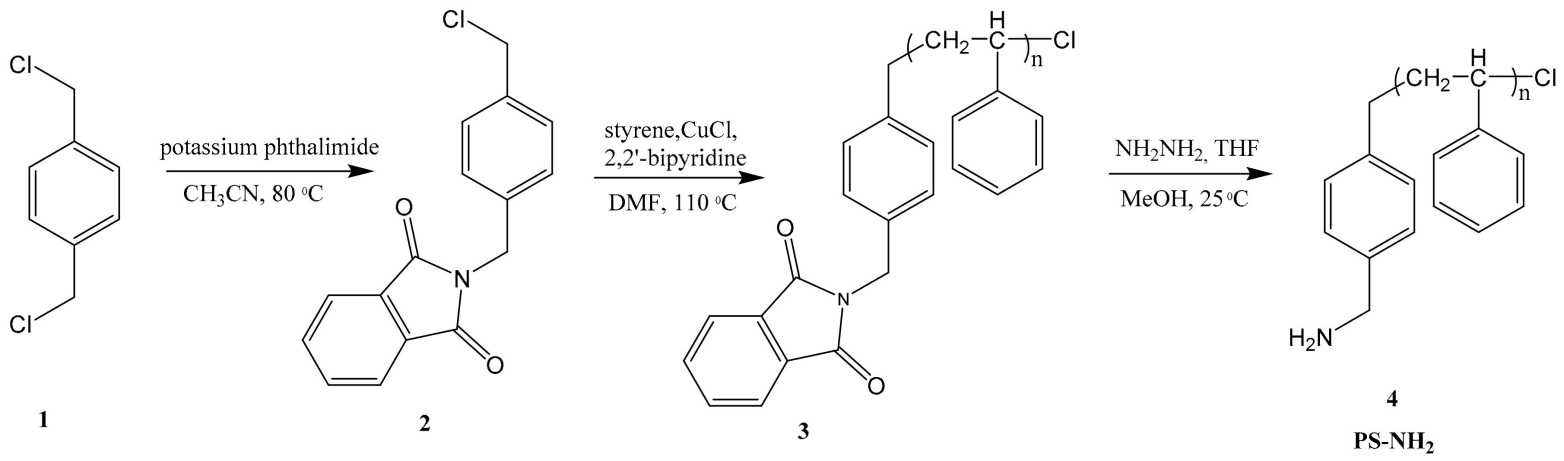
Synthesis route for amine-end-functionalized PS–NH_2_.

In design of a functional polymer for Co NPs, the binding properties to the NP surface should be taken into consideration. It has been proven that surfactants with functional groups –COOH, –NH_2_, and –PO have a strong affinity to the surface of cobalt, since N, O, and P can donate the lone-pair electrons to the empty d-orbitals of the Co atoms [5]. ATRP is the most widely used controlled radical polymerization [34,35,36,37]. The molecular weight, molecular weight distribution, as well as the functionality can be precisely controlled via ATRP technique [38,39,40]. Furthermore, ATRP can be conducted in a variety of solvents, including water, organic solvent, or a mixture thereof. In this study, polystyrene containing a terminal amine group providing anchors for binding to the surface of Co NPs was prepared by using the ATRP technique, similar to the report by Pyun’s group [32]. It was aimed at synthesizing polymers of different molecular weight by varying the amount of monomer and the reaction time. The synthesis process of amine end-functionalized polystyrene (PS-NH_2_) is shown in Scheme 1, which is known as the Gabriel synthesis [41]. Specifically, the primary chloride group in compound 1 reacted with potassium phthalimide to give compound 2. Polymerization of styrene in DMF was initiated by 2, followed by hydrolysis of phthalimide to give amine-ended polystyrene 4. ^1^H-NMR spectra of the intermediate compounds and the final polymer are shown in Figures S1–S3. GPC analysis revealed that PS–NH_2_ with three different molecular weight (number average, *M*_n_) of 2300, 4500 and 10,500 g/mol were synthesized, as shown in Figure 1. Relative broad molecular weight distribution (*M*w/*M*n = 1.38) was observed for PS–NH_2_ with low molecular weight of 2300 g/mol, while, a narrower molecular weight distribution (*M*_w_/*M*_n_ = 1.28) was obtained for PS–NH_2_ with higher molecular weight of 4500 g/mol. The narrowest molecular weight distribution (*M*_w_/*M*_n_ = 1.17) was obtained for polystyrene with the highest molecular weight of 10,500 g/mol. In addition, matrix-assisted laser desorption/ionization time-of-flight (MALDI-ToF) mass spectra were also obtained for PS–NH_2_ (Figure S4).

### 3.2. Synthesis and Characterization of PS–Co NPs

**Figure 2:**
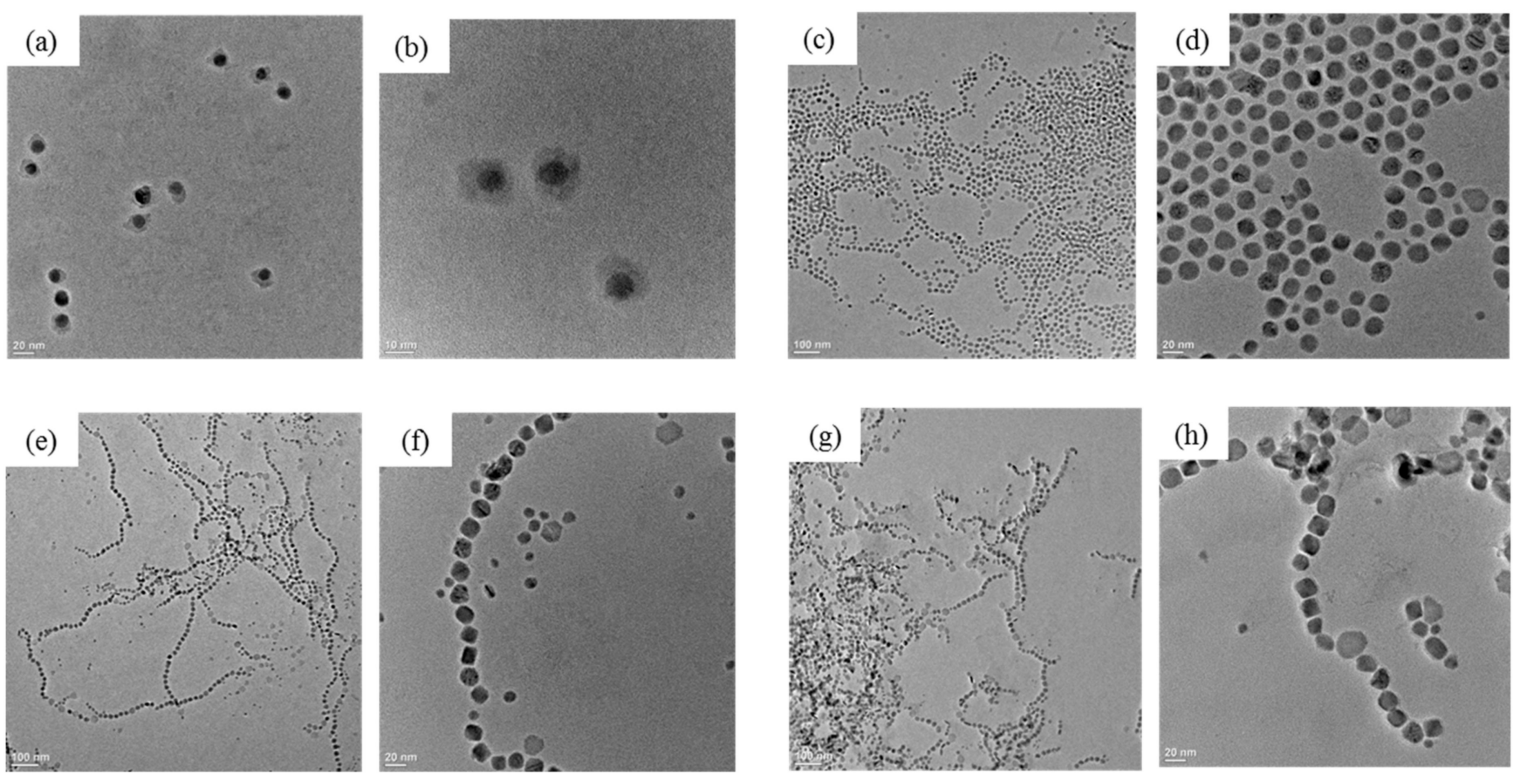
TEM images of PS-Co NPs synthesized with the concentration of cobalt carbonyl fixed at 21.9 mM, and with different concentration of PS–NH_2_ (*M*_n_ = 2300 g/mol, *C*_PS_): (**a**,**b**) *C*_PS_ = 5 mM, *D* = 12.0 ± 1.9 nm; (**c**,**d**) *C*_PS_ = 2.5 mM, *D* = 20.7 ± 1.3 nm; (**e**,**f**) *C*_PS_ = 1.25 mM, *D* = 17.8 ± 2.1 nm; (**g**,**h**) *C*_PS_ = 0.63 mM, *D* = 19.7 nm ± 3.3 nm. The PS–Co NP samples were prepared on TEM grids from respective Co NP dispersion in DCB. The mean size and standard deviation were obtained from a sample of around 200 NPs using ImageJ software.

**Figure 3:**
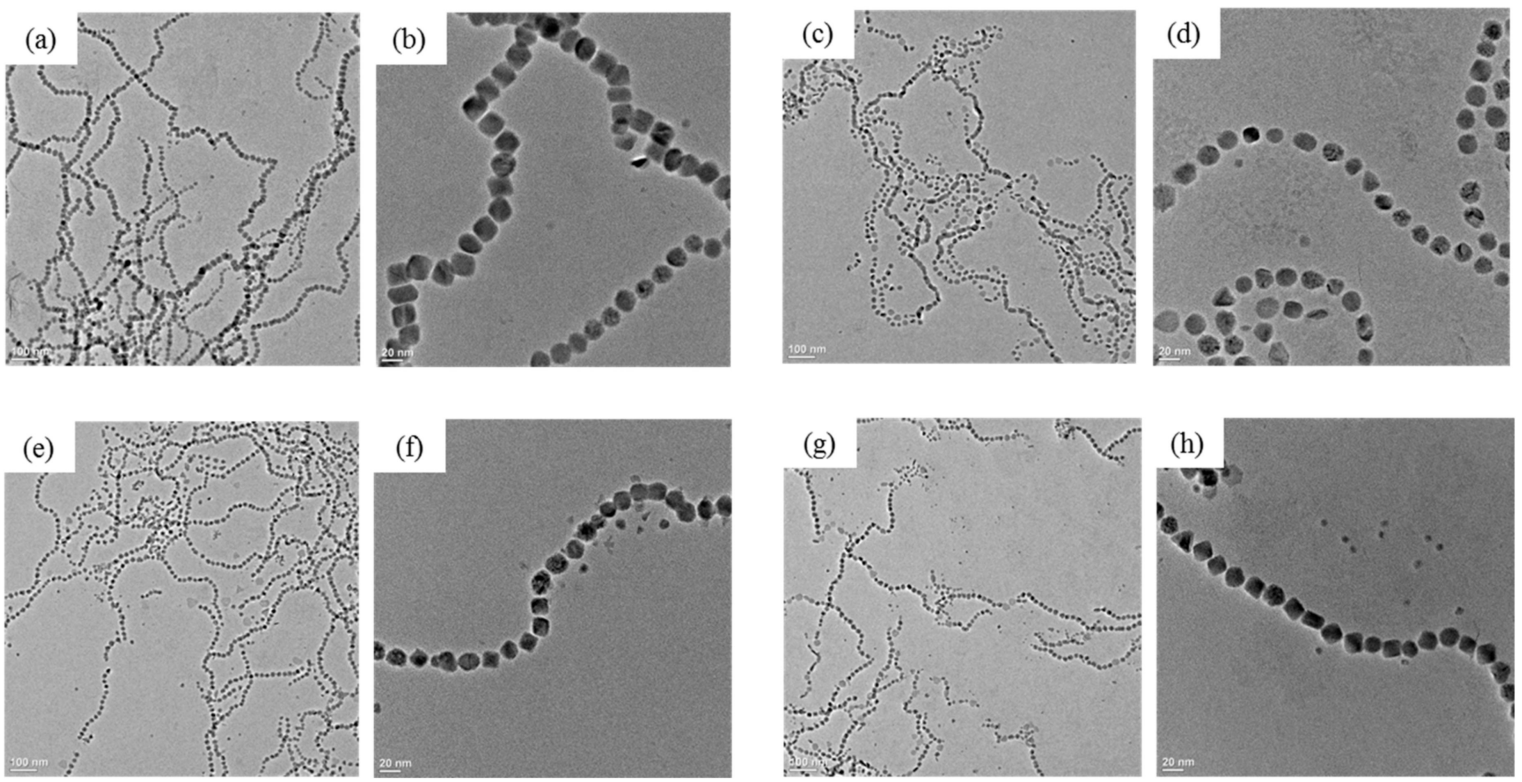
TEM images of PS-Co NPs synthesized with the concentration of cobalt carbonyl fixed at 21.9 mM, and with different concentration of PS-NH_2_ (*M*_n_ = 4500 g/mol, *C*_PS_): (**a**,**b**) *C*_PS_ = 5 mM, *D* = 21.4 ± 1.9 nm; (**c**,**d**) *C*_PS_ = 2.5 mM, *D* = 20.4 ± 2.1 nm; (**e**,**f**) *C*_PS_ = 1.25 mM, *D* = 19.5 ± 1.5 nm; (**g**,**h**) *C*_PS_ = 0.63 mM, *D* = 18.4 ± 3.0 nm. The PS–Co NP samples were prepared on TEM grid from the respective NP dispersion in DCB. The mean size and standard deviation were obtained from a sample of around 200 NPs using ImageJ software.

**Figure 4:**
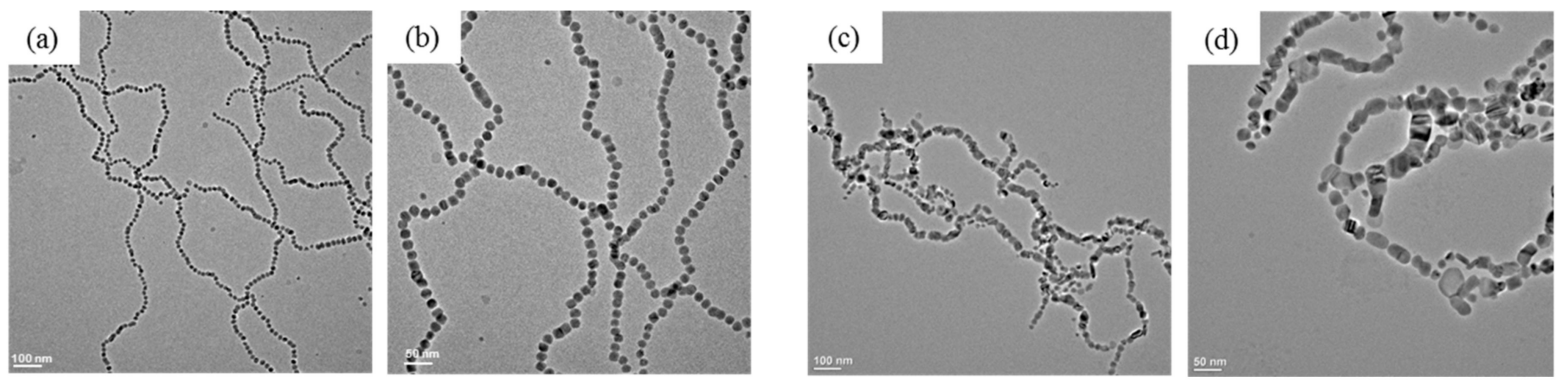
TEM images of PS–Co NPs synthesized with the concentration of PS–NH_2_ (*M*_n_ = 4500 g/mol) at 2.5 mM, only with different concentration of cobalt carbonyl (C_Co_): (**a**,**b**) *C*_Co_ = 7.3 mM, *D* = 18.8 ± 1.3 nm; (**c**,**d**) C_Co_ = 58.4 mM, *D* = 26.6 ± 7.9 nm. The PS–Co NP samples were prepared on TEM grid from the respective NP dispersion in DCB. The mean size and standard deviation were obtained from a sample of around 200 NPs using ImageJ software.

**Figure 5:**
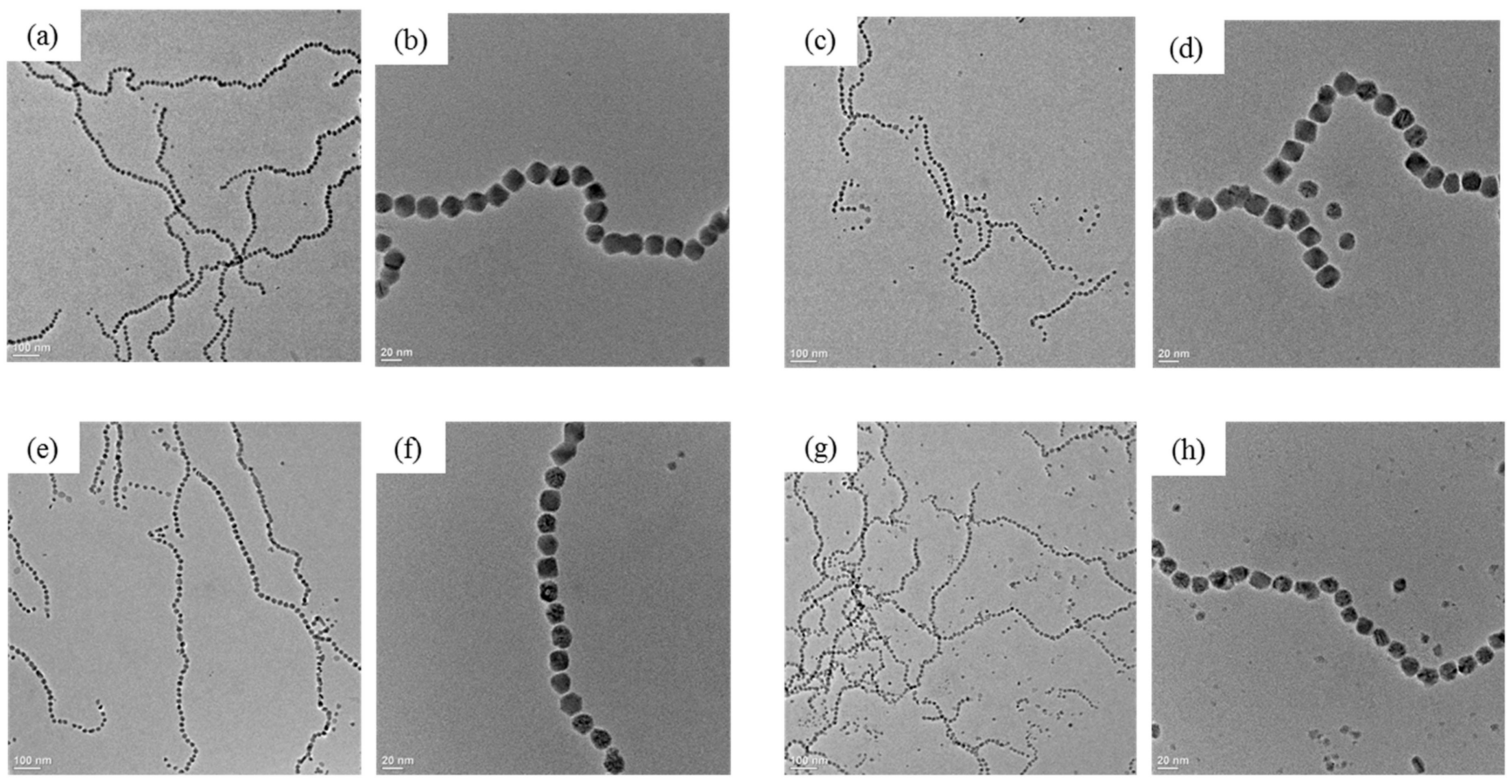
TEM images of PS–Co NPs synthesized with the concentration of cobalt carbonyl fixed at 21.9 mM, and only with different concentration of PS–NH_2_ (*M*_n_ = 10,500 g/mol, *C*_PS_): (**a**,**b**) *C*_PS_ = 5 mM, *D* = 20.0 ± 2.0 nm; (**c**,**d**) *C*_PS_ = 2.5 mM, *D* = 20.5 ± 1.7 nm; (**e**,**f**) C_PS_ = 1.25 mM, *D* = 21.6 ± 1.6 nm; (**g**,**h**) C_PS_ = 0.63 mM, *D* = 16.8 ± 1.8 nm. The PS–Co NPs dispersion in DCB was dropped on to the TEM grid. The mean size and standard deviation were obtained from a sample of around 200 NPs using ImageJ software.

**Figure 6:**
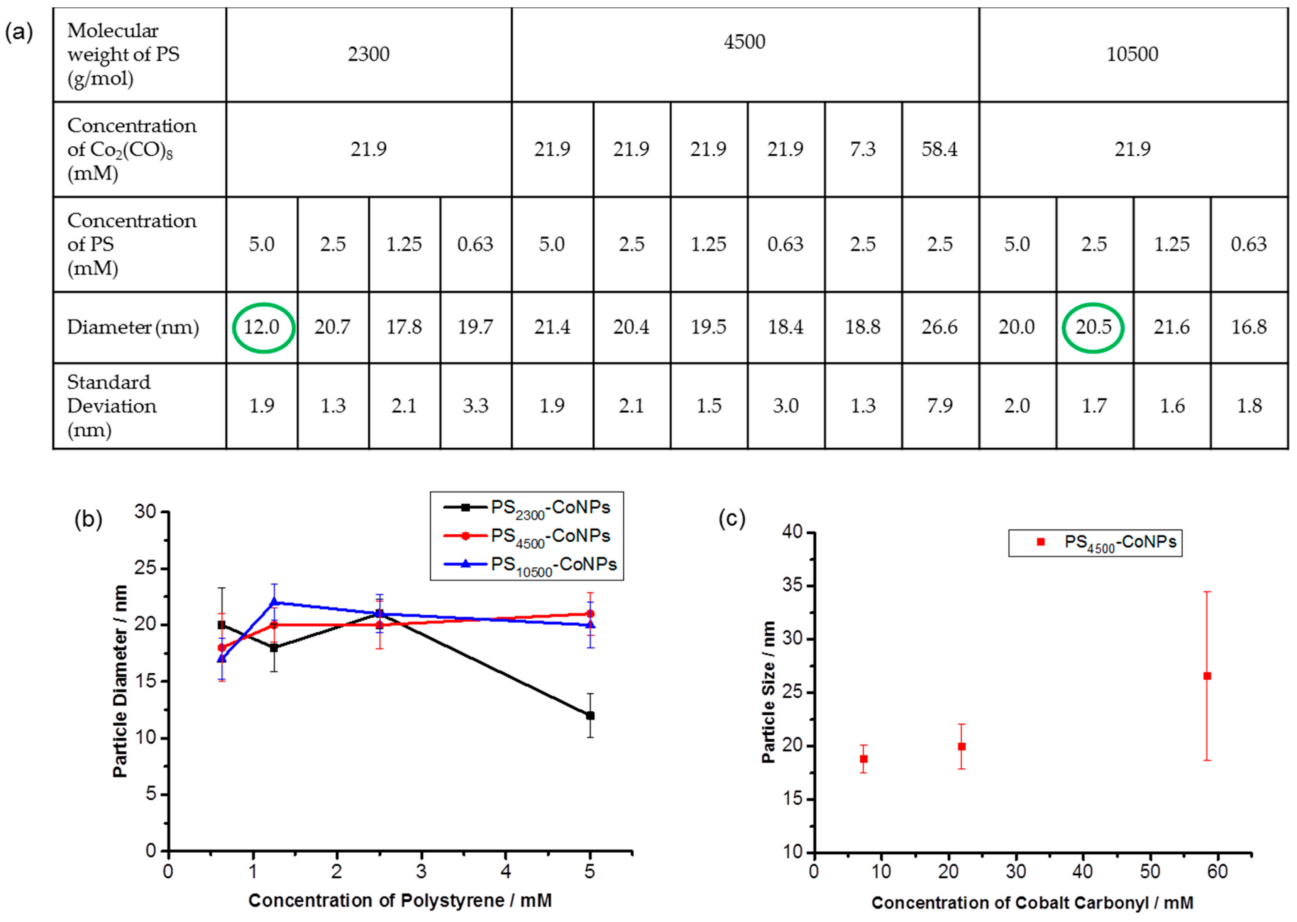
(**a**) Overview of varied synthesis parameters and mean diameters of the as-synthesized PS–Co NPs; (**b**) plot of size distribution of PS–Co NPs synthesized by fixing the concentration of cobalt carbonyl at 21.9 mM, the concentration and molecular weight of PS–NH_2_ was varied; (**c**) plots of size distribution of PS–Co NPs synthesized by fixing the concentration of PS–NH_2_ (2.5 mM, *M*_n_ = 4500 g/mol), while varying the concentration of cobalt carbonyl (*C*_Co_). The mean size and standard deviation were obtained from a sample of around 200 NPs using ImageJ software.

Pyun’s group reported that ferromagnetic PS–Co NPs with a size of around 21 nm were obtained by using PS–NH_2_ as surfactant via a dual-stage thermolysis of cobalt carbonyl in 1,2-dichlorobenzene [32]. It is well known that surfactants play an important role in the growth of Co NPs, and thus affect the size of Co NPs. Here, PS–Co NPs of different sizes were prepared by using the same method, but by varying the synthesis parameters with different molecular weight and concentration of PS–NH_2_ surfactants, as well as different concentration of cobalt carbonyl. The mean size and morphology of the as-prepared PS–Co NPs were characterized by TEM. In addition, HR-TEM images of PS–Co NPs could be obtained and are shown in Figures S5–S7. Figure 2 shows NPs synthesized with PS–NH_2_ of 2300 g/mol when varying the polymer concentration (5 mM, 2.5, 1.25, 0.63 mM). Figure 3, Figure 4 and Figure 5 illustrate similar experiments with polymer surfactants of 4500 and 10,500 g/mol, respectively. While the concentration of cobalt carbonyl was fixed at 21.9 mM during the mentioned synthesis approaches, it was also varied (7.3 mM, 58.4 mM) for a given polymer system (4500 g/mol, 2.5 mM), as shown in Figure 4. Figure 6a presents a tabular overview of the size and size distribution of all prepared nanoparticles, the mean size and standard deviation were obtained from a sample of around 200 NPs using ImageJ software fitting the clearly analyzable particles. Some images show aggregated ill-shaped particles, which are very difficult to measure, and thus couldn’t be taken into account. Therefore, the standard deviation could be slightly underestimated for some samples. Figure 6a,b show that the molecular weight of the polymer has little influence on the size of the particles. Only in the case of low molecular weight and high concentration of PS, the particle size is significantly smaller. This difference in size is very interesting as it changes the magnetic properties of the nanoparticles, as will be discussed later.

Figure 2 demonstrates that with the same molecular weight of PS-NH_2_ (*M*_n_ = 2300 g/mol) and a fixed concentration of cobalt carbonyl (21.9 mM), only by changing the concentration of PS–NH_2_, the size of PS–Co NPs varied significantly, ranging from 12 to 21 nm. The morphology of the PS-Co NPs was also different. With higher concentration of PS–NH_2_, the particles were well-defined, smaller in size, and spherical in shape. However, with lower concentration of the polymer, the particles are larger, ill-defined, and had different shapes. It is interesting to note that the particles were surrounded by a thick polymer layer on the surface when using a higher polymer concentration (5 mM). In addition, they were clearly separated from each other (Figure 2a,b), which results from their superparamagnetic properties (as will be reported in Section 3.3). However, with lower polymer concentration of 2.5 mM, the distance between the particles was shorter (Figure 2c,d). When the concentration of the polymer was lowered to 1.25 mM, the distance between the particles was further reduced and 1-D chain structures were observed (Figure 2e,f). When the polymer concentration further reduced (0.67 mM), the NPs were polydispersed and aggregated as the polymer could not protect the particles anymore (Figure 2g,h). Similarly, Figure 6a also shows that the lower the polymer concentration, the more polydispersed the particles are. 

PS–Co NPs with higher molecular weight of PS–NH_2_ (*M*_n_ = 4500 g/mol) as surfactant were prepared next. On the contrary to the synthesis of PS–Co NPs with PS–NH_2_ (*M*_n_ = 2300 g/mol), the size of the nanoparticles did not change notably by varying the concentration of the polymer. Most of these nanoparticles were around 20 nm in size and formed 1-D chain structures (Figure 3a–h). Furthermore, the influence of the concentration of Co_2_(CO)_8_ on the size and size distribution of PS-Co NPs was also investigated. The concentration of polymer was fixed at 2.5 mM for these experiments. Well-defined and narrowly distributed particles were obtained when the concentration of cobalt carbonyl is lower (7.3 mM) than the usually used concentration (21.9 mM) (Figure 4a,b). When increasing the concentration of Co_2_(CO)_8_ up to 58.4 mM, ill-defined particles were observed (Figure 4c,d). Figure 6c revealed that the higher concentration of Co_2_(CO)_8_, which means a lower ratio of polymer/cobalt, resulted in larger and polydispersed PS–Co NPs. Thus, to obtain Co NPs with a well-defined shape, a ratio of PS–NH_2_/Co_2_(CO)_8_ greater than 0.1 is required.

PS-Co NPs were synthesized with a third molecular weight of PS–NH_2_ (*M*_n_ = 10,500 g/mol) as surfactant. Similar to the NPs prepared with PS–NH_2_ (*M*_n_ = 4500 g/mol), the size of the nanoparticles was around 20 nm and did not change significantly by varying the concentration of the polymer. Most of these nanoparticles self-assembled into 1-D chain structures (Figure 5). Figure 6b shows the size distribution when using different molecular weight and concentration of PS–NH_2_. A significant change in the size was observed only with the molecular weight of 2300 g/mol and the concentration of 5 mM by fixing cobalt carbonyl concentration at 21.9 mM. It has been reported that the ratio of stabilizer-to-precursor can affect the size of nanoparticles, and the dependence can be reverse due to different mechanisms of particles growth [42]. For example, the size of iron oxide nanocrystals stabilized by oleic acid increased with the ratio of oleic acid to Fe(CO)_5_ [43]. However, the size of gold nanoparticles decreases with the ratio of thiol to precursor [44]. Multiple mechanisms of nucleation and growth of nanoparticles exist, and different mechanisms will lead to different size and morphology of the nanoparticles [45,46,47,48,49]. One possible reason for the observation that Co NPs synthesized with PS–NH_2_ (*M*_n_ = 2300 g/mol) at higher concentration were smaller than those synthesized in the presence of PS of higher molecular weight could be derived from the Co NPs growth process and the steric hindrance of the surfactant. After injection of metal precursor Co_2_(CO)_8_, nucleation takes place rapidly, followed by the growth of the nuclei. After nucleation, the solution contains nuclei, Co-surfactant complexes, and free Co atoms. Because of the long chain of higher molecular weight PS, the Co-surfactant complexes have a smaller diffusion coefficient, which will result in lower monomer reactivity, thus fewer nuclei are produced in the solution. Therefore, there will be high monomer concentration to keep the same flux of monomers in the solution, which leads to the formation of larger particles [6,46]. The term “monomer” refers to molecular precursors which participate in the reversible behavior of adding/removing the Co atoms to a NP [45]. However, the lower molecular weight of PS–NH_2_ with less steric hindrance has a higher diffusion coefficient of Co-surfactant complexes and thus a higher monomer reaction rate, which results in a high concentration of nuclei. Consequently, monomers will quickly be depleted, which leads to the formation of smaller nanoparticles [6]. Apart from the explanation mentioned above, another reason would also need to be taken into consideration. A higher fraction of polymer would be attached to the surface of a NP at a higher concentration of polymer, which would prevent further growth of the NP. Thus, NPs synthesized with low molecular weight PS (*M*_n_= 2300 g/mol) at higher concentration formed smaller NPs than those at lower concentrations. However, this is only a hypothesis and carefully designed experiments and computer simulations are needed to prove the growth process of PS-Co NPs. 

In view of the difference in size and morphology between PS–Co NPs prepared by varying the parameters, two particles were exemplarily chosen to compare their magnetic properties, crystalline structures, and grafting density. The first sample is PS–Co NPs of comparably small size of 12.0 nm prepared from PS–NH_2_ (*M*_n_ = 2300 g/mol, 5 mM; PS_2300_–Co NPs), and the second is the NPs of 20.5 nm prepared from PS–NH_2_ (*M*_n_ = 10,500 g/mol, 2.5 mM; PS_10500_–Co NPs), which are marked with green circles in Figure 6a. The NPs were prepared with cobalt carbonyl concentration of 21.9 mM, thus the main difference in synthesis originated from the molecular weight and concentration of the polymeric surfactant.

### 3.3. Magnetic Properties of PS-Co NPs

**Figure 7:**
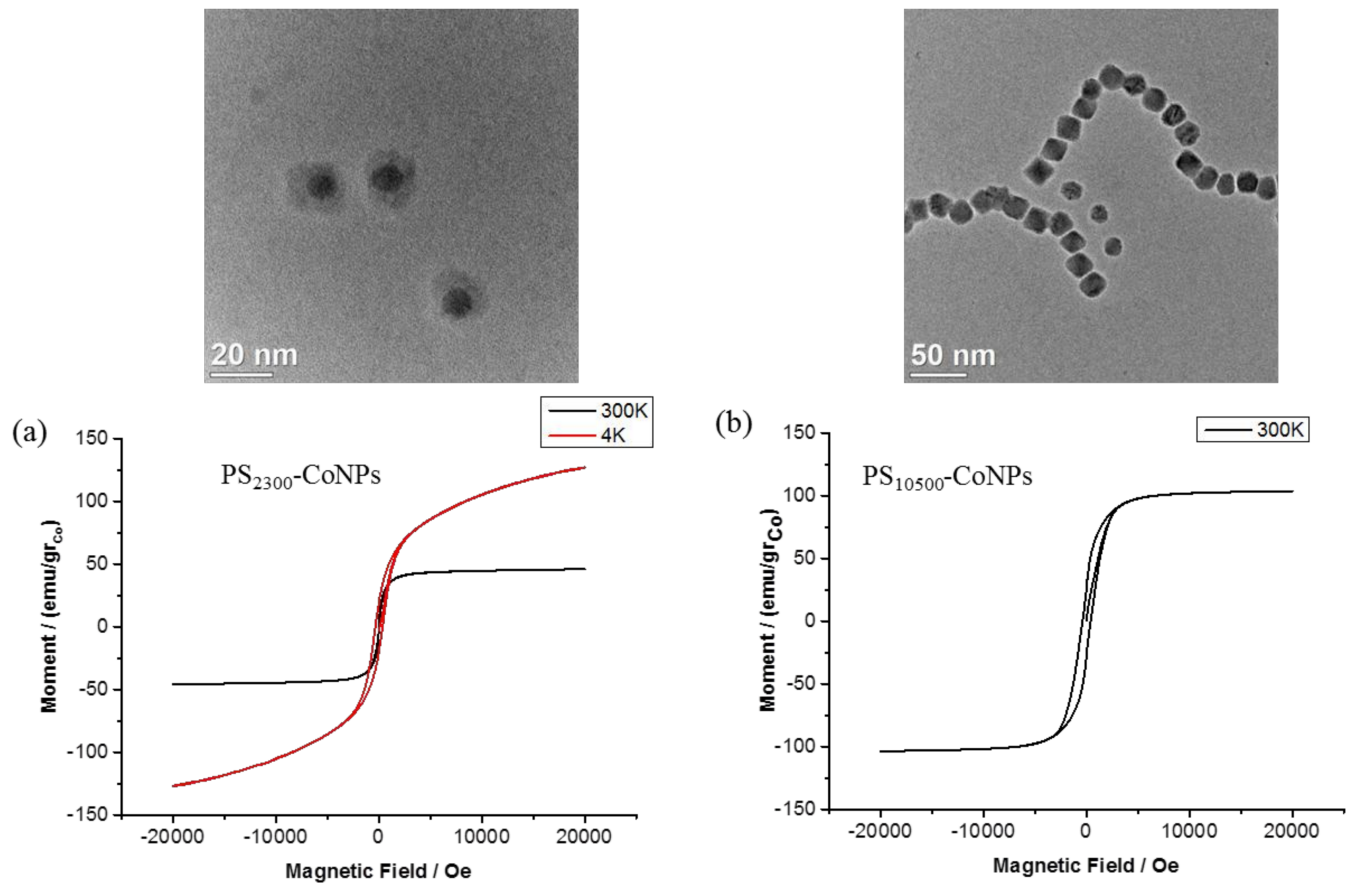
TEM images (**upper**) and field dependence of magnetization of PS–Co NPs (**lower**): (**a**) PS_2300_–CoNPs; (**b**) PS_10500_–Co NPs.

The measurements were conducted by using a SQUID magnetometer. TEM images and magnetization curves of the two nanoparticles are shown in Figure 7. The magnetic behavior of the PS_2300_–Co NPs was characterized by a hysteresis loop at 4 K (Figure 7a). At 300 K, no hysteresis appears, verifying the expected superparamagnetic behavior of the synthesized particles with a size below the critical diameter for Co NPs (12.0 nm) [50]. No saturation magnetization was observed at 4 K due to the thick polystyrene layer on the surface of Co NPs, which were shown in TEM images (Figure 2a,b). The magnetization of the PS_2300_–Co NPs is about 125 emu/g_Co_ at 4 K and 45 emu/g_Co_ at 300 K, which are lower than the saturation magnetization of bulk Co (162 emu/g) at room temperature [51,52,53]. The reason for this small reduction could be manifold. It is well-known that the energy of a magnetic nanoparticle under an external magnetic field is proportional to the volume of the particle, thus the energy of nanoparticles is also small, and the thermal fluctuation greatly reduces the total magnetic moment [54]. Furthermore, the thick layer of polystyrene bound to the surface of the nanoparticles can also quench the magnetic moment [55]. Last but not least, the magnetic cobalt atoms on the surface of the nanoparticles, which have an incomplete coordination that increases their spin disorientation, can further decrease the effective magnetic moment [56,57,58,59]. While the 12.0 nm NPs are superparamagnetic, a hysteresis loop appeared for the PS_10500_–Co NPs at 300 K, which indicated the ferromagnetic behavior of these 20.5 nm particles. The saturation magnetization of PS_10500_–Co NPs at 300 K is around 104 emu/gr_Co_, which is higher than that of PS_2300_–Co NPs, indicating permanent dipolar interaction between the nanoparticles. This is the explanation why PS–Co NPs with larger sizes can self-assemble into 1-D chain structures, as shown in TEM images (Figure 7). 

### 3.4. Wide Angle X-ray Scattering of PS-Co NPs

**Figure 8:**
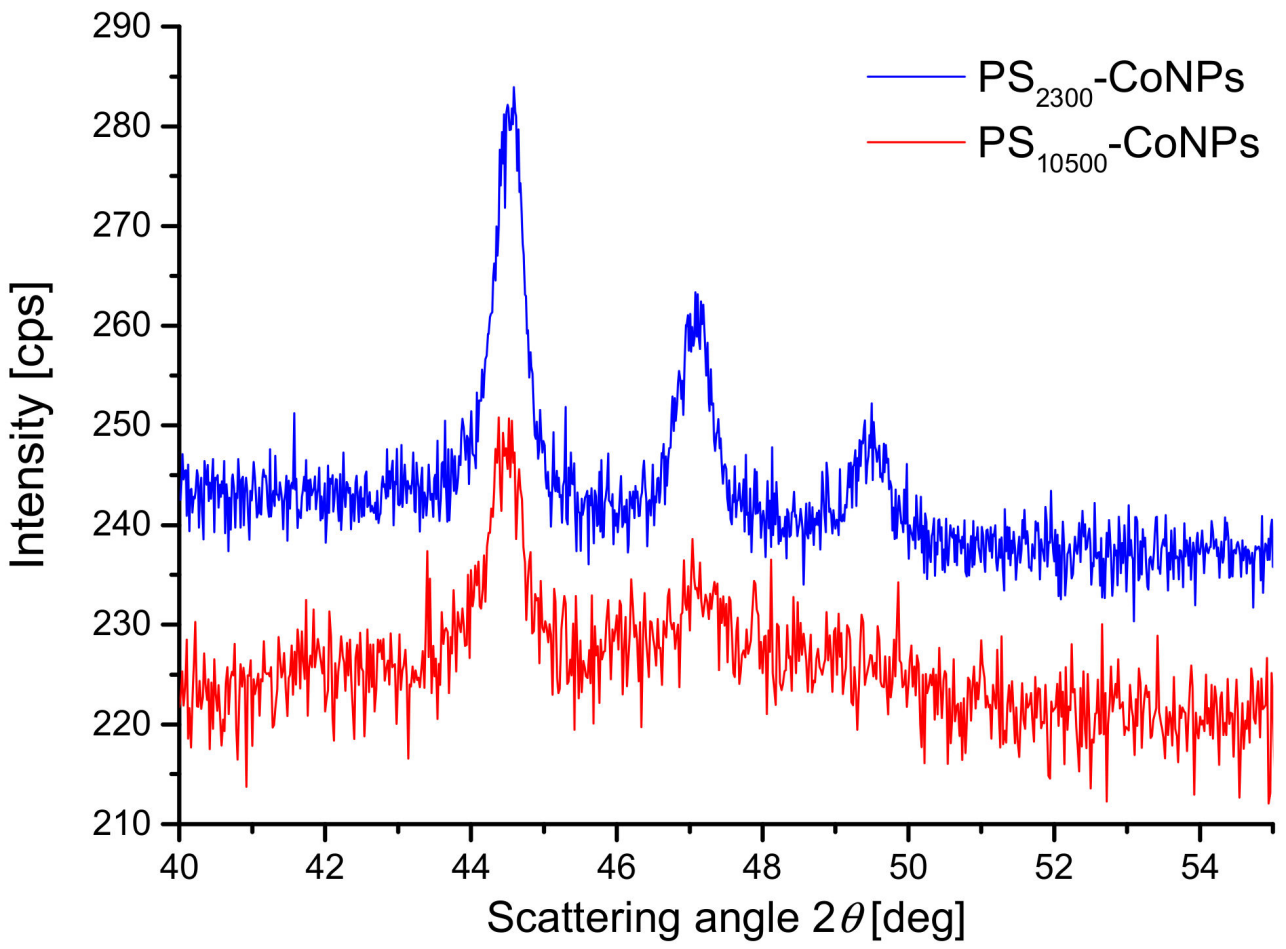
Wide angle X-ray scattering (WAXS) patterns of PS_2300_–Co NPs and PS_10500_–Co NPs.

Cobalt nanoparticles have appeared in two different crystal structures—hexagonal-close-packed (hcp) and face-centered-cubic (fcc). However, when Bawendi et al. synthesized cobalt nanoparticles through the thermolysis of cobalt carbonyl with trioctylphosphane oxide (TOPO) as the ligand, a new crystalline phase, the epsilon (ɛ) structure, was identified [60]. Later, other researchers also found the epsilon cobalt crystalline phase by preparation via decomposition of cobalt carbonyl at high temperature with oleic acid (OA) and dioctylamine (DOA) as surfactants [6]. In this study, wide angle X-ray scattering was used to investigate the crystalline phase of 12.0 nm PS_2300_–Co NPs and 20.5 nm PS_10500_–Co NPs. As shown in Figure 8, both of these two samples exhibited the epsilon (ɛ) crystal structure, the diffraction peaks at 2θ angles 44.6°, 47.1°, and 49.6° are corresponding to (221), (310), and (311) reflections, respectively [6,7,61]. This was in accordance with the previous report by Pyun et al., who synthesized PS–Co NPs by using PS–NH_2_ as surfactant via a dual-stage temperature decomposition of Co_2_(CO)_8_ at 175 and 160 °C [32]. In Figure 8, the sample PS_2300_–Co NPs shows sharper and clearer peaks than that of PS_10500_–Co NPs. Nevertheless, both spectra allow the conclusion that Co NPs are detected in the ɛ-phase. 

### 3.5. Calculation of Surface Polymer Density

Generally, thermogravimetric analysis (TGA) is a widely used technique to evaluate the organic content on the surface of NPs [62,63,64,65,66]. The density of polystyrene on the nanoparticle surface was calculated according to TGA. The equation for calculation of the grafting density of the polymer content on the surface of cobalt nanoparticles (φ) is shown below:

$$\varphi =\frac{N_{\left(polymer\right)}}{N_{\left(NPs\right)}S_{\left(NPs\right)}}=\frac{W_{\left(polymer\right)}N_{A}R\rho }{3M_{\left(polymer\right)}W_{\left(NPs\right)}}$$

Here, *W*_(*polymer*)_ and *W*_(*NPs*)_ present the weight loss percentage of polystyrene and the weight percentage of Co NPs, *N*_(*polymer*)_ and *N*_(*NPs*)_ present the number of polystyrene and Co NPs, respectively, *S*_(*NPs*)_ is the total surface area of NPs, ρ is the density of Co NPs (8.9 g/cm^3^), *N_A_* is Avogadro’s number, *R* is the radius of PS–Co NPs, and *M*_(*polymer*)_ is the molecular weight of polystyrene. In this study, the grafting density of 12.0 nm PS_2300_–Co NPs and 20.5 nm PS_10500_–Co NPs was calculated.

Gel permeation chromatography (GPC) and matrix-assisted laser desorption/ionization time-of-flight (MALDI-ToF) mass spectrometry are two popular techniques for the determination of the molecular weight of a polymer. The polymer with lower molecular weight showed *M*_n_ of 3130 Da according to the MALDI-ToF measurement (Figure S4a), which was higher than the 2300 g/mol measured by GPC (Figure 1). However, the molecular weight of 10,600 Da measured by MALDI-ToF mass spectrometry for the second polymer, was in agreement with the 10,500 g/mol measured by GPC. In fact, the molecular weight determined by MALDI-ToF is similar to the GPC value only if the polymer has a narrow molecular weight distribution (*M*_w_/*M*_n_ < 1.2) [67,68]. For broad distributions, the molecular weight obtained by MALDI-ToF mass spectrometry usually deviates from the real molecular weight. Since the value of *M*_w_/*M*_n_ is 1.17 for PS (*M*_n_ = 10,500 g/mol), which is lower than 1.2, the molecular weight measured by MALDI-ToF matches with GPC. However, for PS (*M*_n_ = 2300 g/mol), the value of *M*_w_/*M*_n_ is 1.38, which is beyond the range of realistic values obtained by MALDI-ToF, and thus the deviation between the results of these two techniques is not surprising. Therefore, the molecular weight of the polymers measured by GPC was chosen for the calculation of the grafting density. In addition to the above arguments, it can be expected that the GPC results should be rather accurate because polystyrene was used for calibration during GPC measurements.

**Figure 9:**
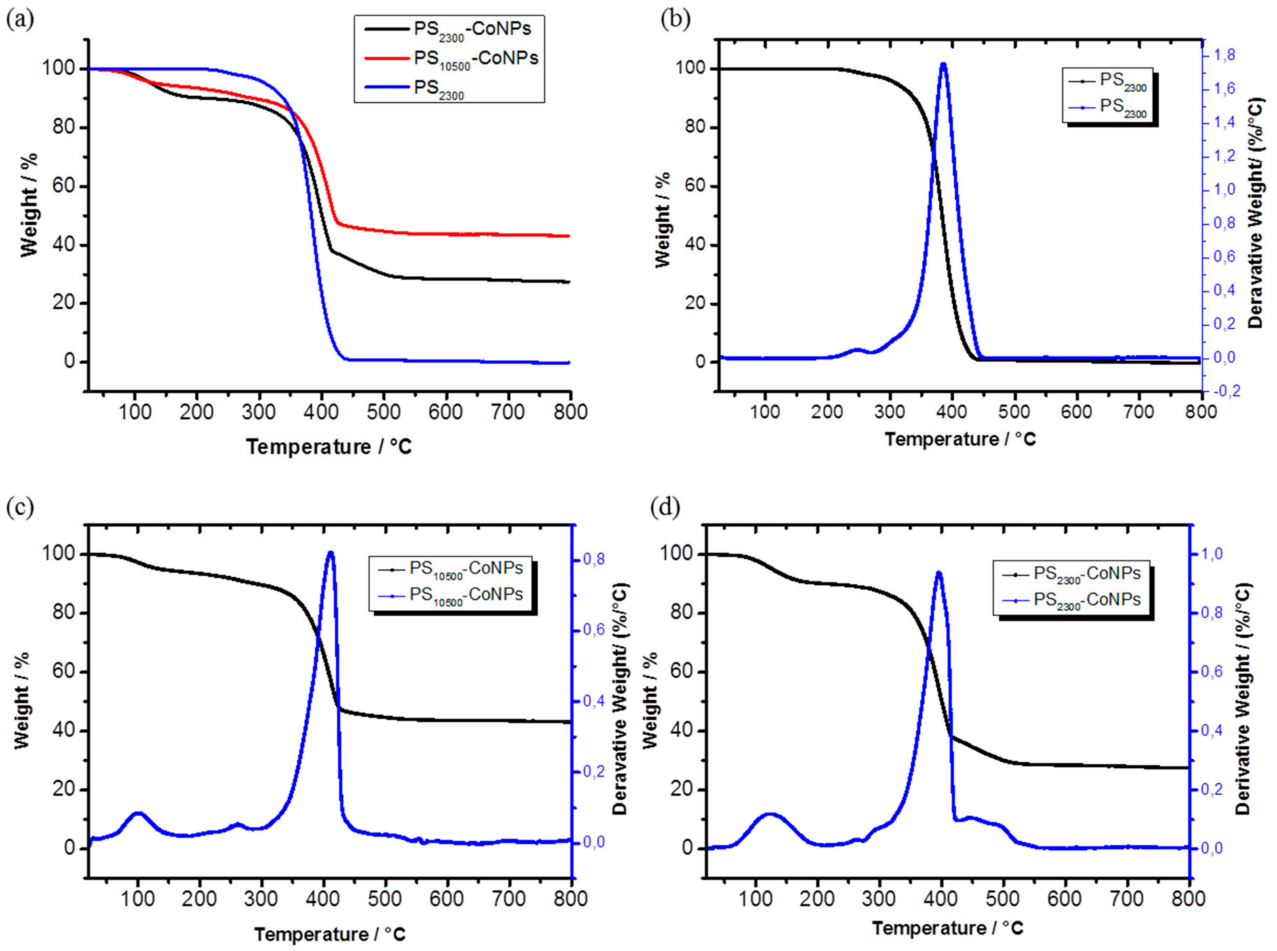
TGA analysis: (**a**) overview profile of PS_2300_–Co NPs, PS_10500_–Co NPs and free PS–NH_2_ (*M*_n_ = 2300 g/mol); (**b**) free PS–NH_2_ (*M*_n_ = 2300 g/mol); (**c**) PS_10500_–Co NPs; (**d**) PS_2300_–Co NPs. The black lines indicate the percent weight as a function of temperature and the weight loss derivative is shown in blue.

For the TGA measurement, both samples were washed twice by adding a small amount of hexane to flocculate PS–Co NPs, followed by centrifugation and drying in vacuum oven for two days. The TGA overview shows the weight loss as a function of temperature for the Co NP samples with the two investigated polymers as well as for free PS–NH_2_ (*M*_n_ = 2300 g/mol) (Figure 9a). Figure 9b demonstrates that the weight loss in the temperature range of 200–420 °C corresponds to the decomposition of PS–NH_2_. For PS_10500_–Co NPs, the weight loss of about 6.5% below 200 °C is attributed to removal of adsorbed solvent. The significant weight loss stages of 47.5% in total which is observed between 200–420 °C is attributed to the decomposition of PS–NH_2_ (*M*_n_ = 10,500 g/mol) attached to the surface of Co NPs (Figure 9c). Figure 9d shows the TGA profile of PS_2300_–Co NPs. The initial weight loss of about 9.8% below 200 °C is equally explained by adsorbed solvent. The weight loss of about 52.8% in the temperature range of 200–420 °C can be ascribed to the decomposition of PS–NH_2_ (*M*_n_ = 2300 g/mol) on the surface. Therefore, for PS_10500_–Co NPs, with *W*_(*polymer*)_ = 47.5% and *W*_(*NPs*)_ = 43.6%, the polymer density was calculated to 1.9 chains/nm^2^. Similarly, the polymer density was calculated for PS_2300_–Co NPs, with *W*_(*polymer*)_ = 52.8% and *W*_(*NPs*)_ = 28%, as 8.7 chains/nm^2^. This grafting density is even higher than what we obtained previously for poly(*N*-isopropylacrylamide)-*g*-Co NPs via a grafting-from approach [8]. It should be noted that a higher grafting density would be obtained when free polymer is mixed into the washed hybrid nanoparticles. This could happen from the precipitation of PS due to the addition of hexane into 1,2-dichlorobenze. However, high values of grafting density were indeed reported for other systems before [69,70]. Nevertheless, the polymer density of PS_2300_ on the surface of Co NPs is clearly higher than that of PS_10500_, which can be likewise observed in TEM images (Figure 2a,b). One possible reason could arise from the lower steric hindrance of PS_2300_, and hence a higher diffusion coefficient of Co-polymer complexes. A higher fraction of polymer would be attached to the surface of a NP at a higher concentration of polymer, which would prevent further growth of the NP and form a closely packed shell. 

### 3.6. Stability Study

**Figure 10:**
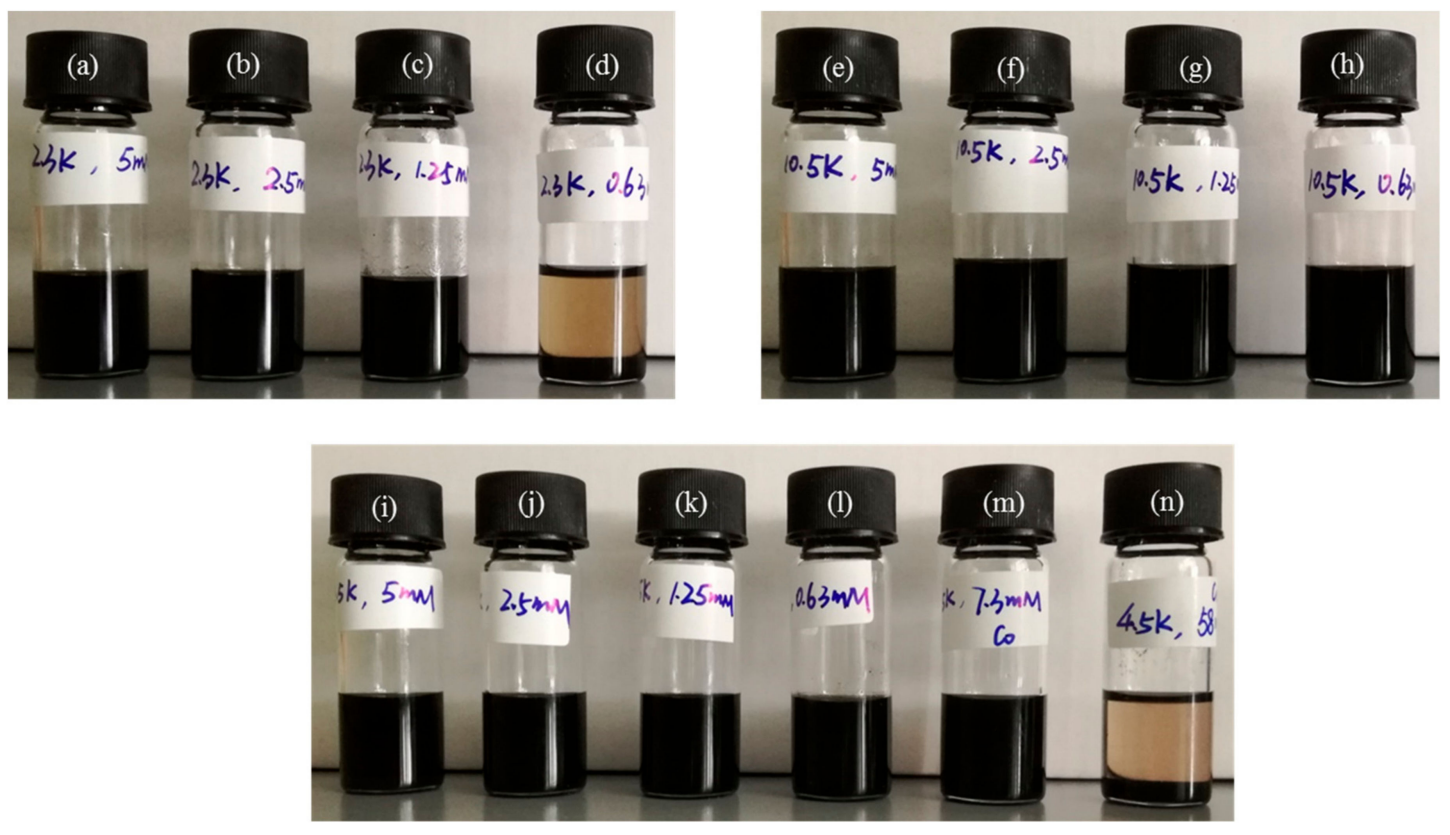
Photographs of PS–Co NPs synthesized at different conditions in 1,2-dichlorobenzene: (**a**–**d**) prepared with PS–NH_2_ (*M*_n_ = 2300 g/mol) at concentration of 5 mM (**a**), 2.5 mM (**b**), 1.25 mM (**c**), 0.63 mM (**d**) with cobalt carbonyl concentration of 21.9 mM; (**e**–**h**) prepared with PS-NH_2_ (*M*_n_ = 10,500 g/mol) at concentration of 5 mM (**e**), 2.5 mM (**f**), 1.25 mM (**g**), 0.63 mM (**h**) with cobalt carbonyl concentration of 21.9 mM; (**i**–**l**) prepared with PS–NH_2_ (*M*_n_ = 4500 g/mol) at concentration of 5 mM (**i**), 2.5 mM (**j**), 1.25 mM (**k**), 0.63 mM (**l**) with cobalt carbonyl concentration of 21.9 mM; (**m**,**n**) prepared with PS–NH_2_ (*M*_n_ = 4500 g/mol) at concentration of 2.5 mM, and cobalt carbonyl concentration of 7.3 mM (**m**) and 58.4 mM (**n**). These photographs were taken three weeks after the synthesis.

The amine end-functionalized polystyrene provides steric repulsion between cobalt nanoparticles and prevents them from agglomerating. As the surfactants play a significant role in the growth of the nanoparticles, their preparation by changing the conditions of surfactant could influence their stability. Therefore, the stability of the NP dispersions was monitored after 20 days. As shown in Figure 10, the stability of the PS–Co NPs can be readily observed visually. Figure 10a–d shows PS–Co NPs prepared by using PS–NH_2_ (*M*_n_ = 2300 g/mol), (Figure 10e–h) PS–NH_2_ (*M*_n_ = 10,500 g/mol), (Figure 10i–n) PS–NH_2_ (*M*_n_ = 4500 g/mol) at different polymer concentrations, respectively. The first conclusion was that Co NPs prepared with higher polymer molecular weight were more stable than these with lower molecular weight at the same concentration. This can be easily observed from the samples with a rather low polymer concentration of 0.63 mM (Figure 10d,h,l), in which (Figure 10d) showed clear precipitation, while Figure 10h and l showed less aggregation or no aggregation. Second, a higher concentration of polymer enhances the stability of the nanoparticles, which can be observed from (Figure 10a) to (Figure 10d), as the nanoparticles gradually become less stable with lower polymer concentration. Third, higher concentration of cobalt carbonyl reduces the stability, which is shown in (Figure 10m,n). All in all, a higher polymer concentration and longer polymer chain (e.g., *M*_n_ = 10,500 g/mol) enhanced the NP stability, which could be stored for at least three months without pronounced aggregation.

## 4. Conclusions

In summary, amine end-functionalized polystyrene-coated cobalt nanoparticles were prepared via the dual-stage thermolysis of cobalt carbonyl (Co_2_(CO)_8_). While several examples for the synthesis of polymer-functionalized magnetic nanoparticles have been reported, we performed a detailed study on the influence of the molecular weight and concentration of the polymeric surfactant as well as the concentration of the metallic precursor on the size, size distribution, grafting density, magnetic properties, and stability of the resulting hybrid particles. The polystyrene surfactants with molecular weight of 2300, 4500, and 10,500 g/mol were prepared via an atom-transfer radical polymerization technique. In general, a low polymer concentration during the synthesis of the hybrid nanoparticles results in ill-defined and polydisperse particles of low stability. The stability of the NPs is ensured by steric repulsion between the polymer chains which prevents agglomeration. Therefore, a higher molecular weight of the polymers enhances the stability of a PS–Co NP dispersion over time. It was found that PS_2300_ with high concentration can lead to smaller particles, which were superparamagnetic. This combination of polymer molecular weight and concentration probably results in a high diffusion coefficient and reactivity of cobalt-polymer complexes, as well as a high fraction of polymer attached to the NPs. In consequence, a small size and a high grafting density were observed for this sample. In contrast, all other experimental conditions led to ferromagnetic NPs around 20 nm in size. In addition to the polymeric surfactant, the concentration of the Co_2_(CO)_8_ precursor was varied and showed that increasing the cobalt carbonyl concentration, which means lowering the polymer/Co ratio, led to large, polydisperse, and unstable NPs. This study provides insights into the synthesis of stable hybrid nanoparticles. In future, such polymer-functionalized cobalt nanoparticles can serve as building blocks for novel mesostructured assemblies.

## Supplementary Materials

The following are available online at http://www.mdpi.com/2073-4360/10/10/1053/s1, Figure S1: ^1^H-NMR spectrum of **2** in CDCl_3_, Figure S2: ^1^H-NMR spectrum of **3** (Mn = 4500 g/mol) in CDCl_3_, Figure S3: ^1^H-NMR spectrum of **4** (Mn = 4500 g/mol) in CDCl_3_, Figure S4: MALDI-ToF mass spectra of polystyrene: 3130 Da (a) and 10,600 Da (b). Figures S5–S7: HR-TEM images of PS-Co NPs.
