# Supplementary material for: Synthesis of Polystyrene-Coated Superparamagnetic and Ferromagnetic Cobalt Nanoparticles
Source: Polymers (Basel). 2018 Sep 20;10(10):1053. doi: 10.3390/polym10101053 (PMC6404081; doi:10.3390/polym10101053)
Supplement: Supplementary file 1 [file polymers-10-01053-s001.pdf]

Supporting Information

Synthesis of Polystyrene-Coated Superparamagnetic and Ferromagnetic Cobalt Nanoparticles

Li Tan [1,2], Bing Liu [3], Konrad Siemensmeyer [4], Ulrich Glebe [1,*], Alexander Böker [1,2,*]

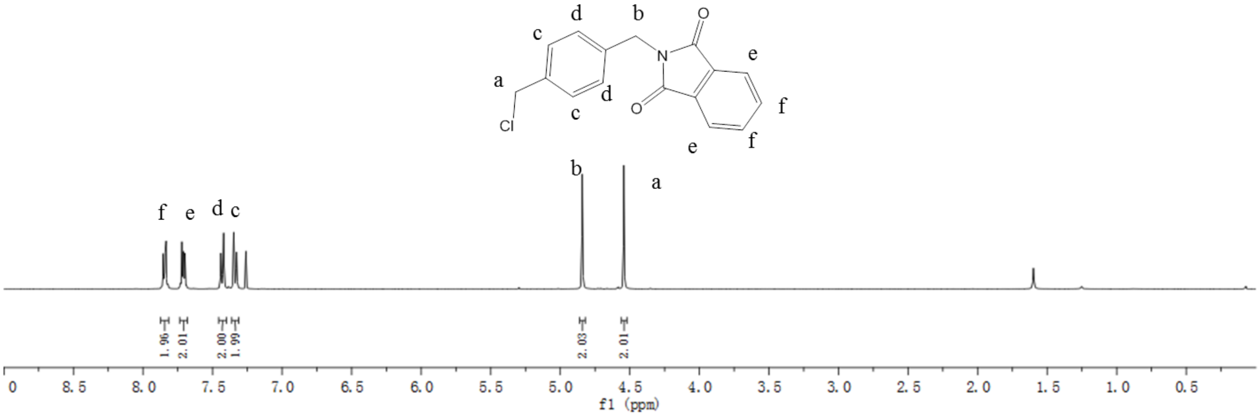

Figure S1. ^{1}H-NMR spectrum of **2** in $CDCl_3$.

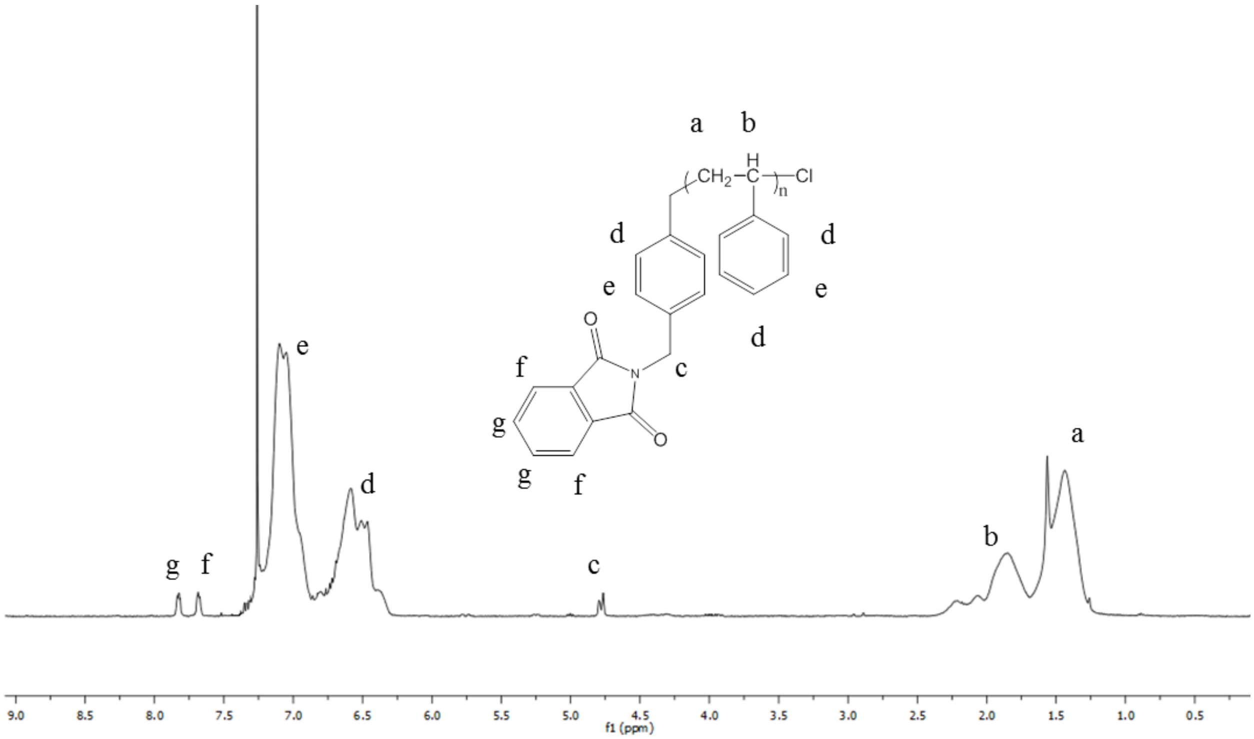

Figure S2. ^{1}H-NMR spectrum of **3** (Mn = 4500 g/mol) in $CDCl_3$.

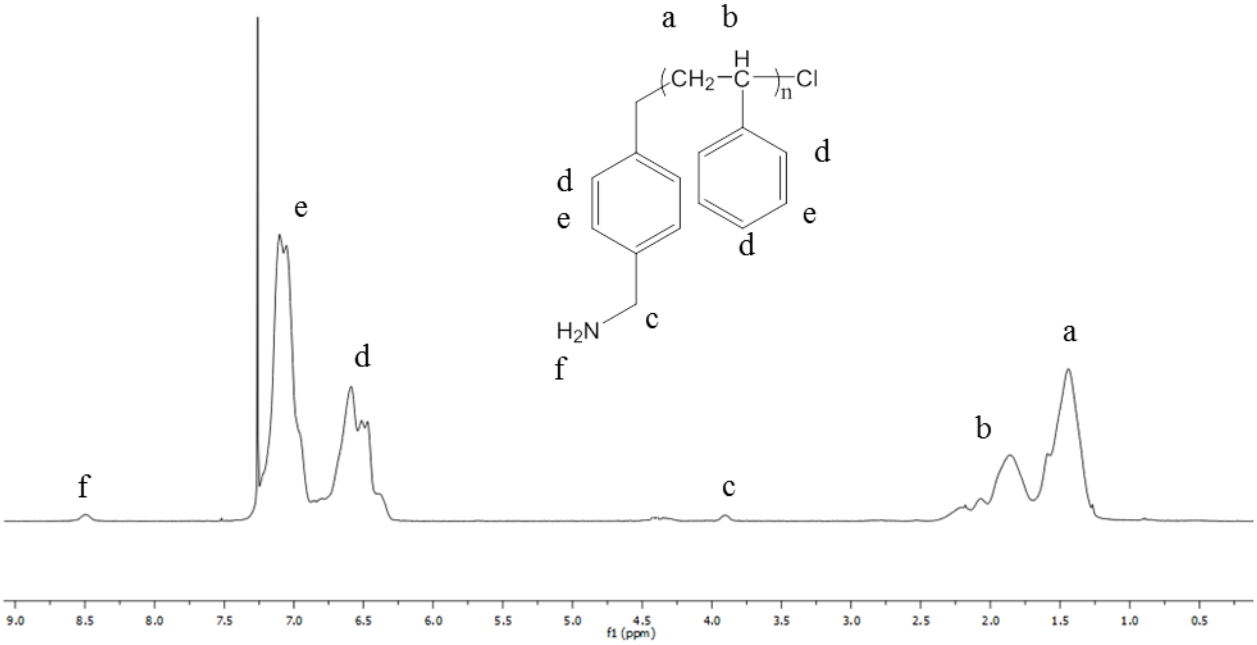

Figure S3. ^{1}H-NMR spectrum of **4** (Mn = 4500 g/mol) in $CDCl_3$.

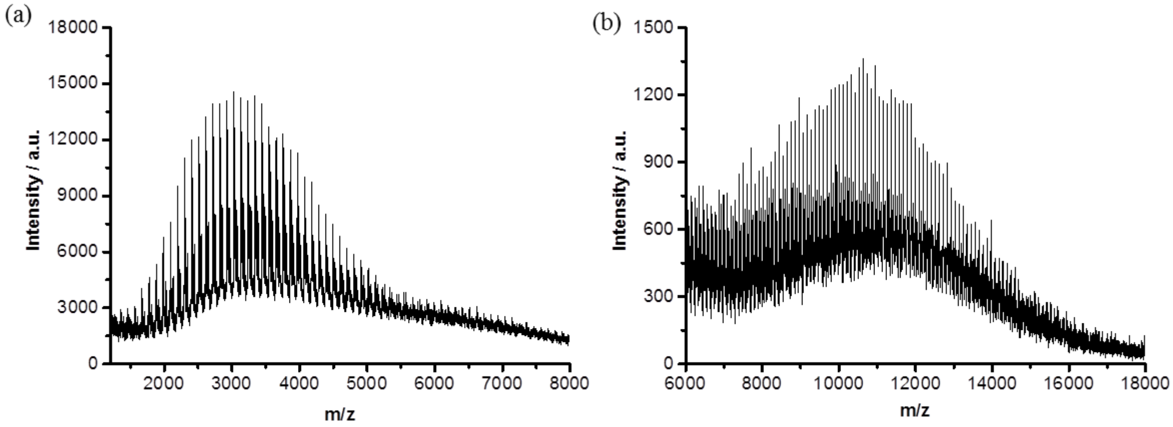

Figure S4. MALDI-ToF mass spectra of polystyrene: 3130 Da (a) and 10600 Da (b).

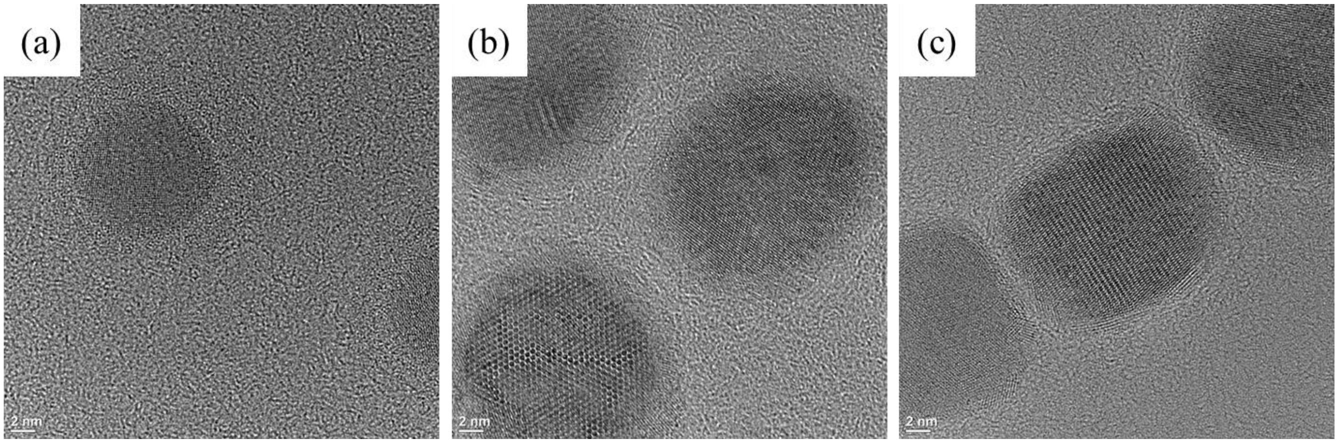

Figure S5. HR-TEM images of PS_{2300}-Co NPs synthesized with the concentration of cobalt carbonyl fixed at 21.9 mM, and with different concentration of $PS\text{-}NH_2$ (Mn = 2300 g/mol, C_{PS}): (a) C_{PS} = 5 mM; (b) C_{PS} = 2.5 mM; (c) C_{PS} = 1.25 mM. The PS-Co NP samples were prepared on TEM grids from respective Co NP dispersion in DCB.

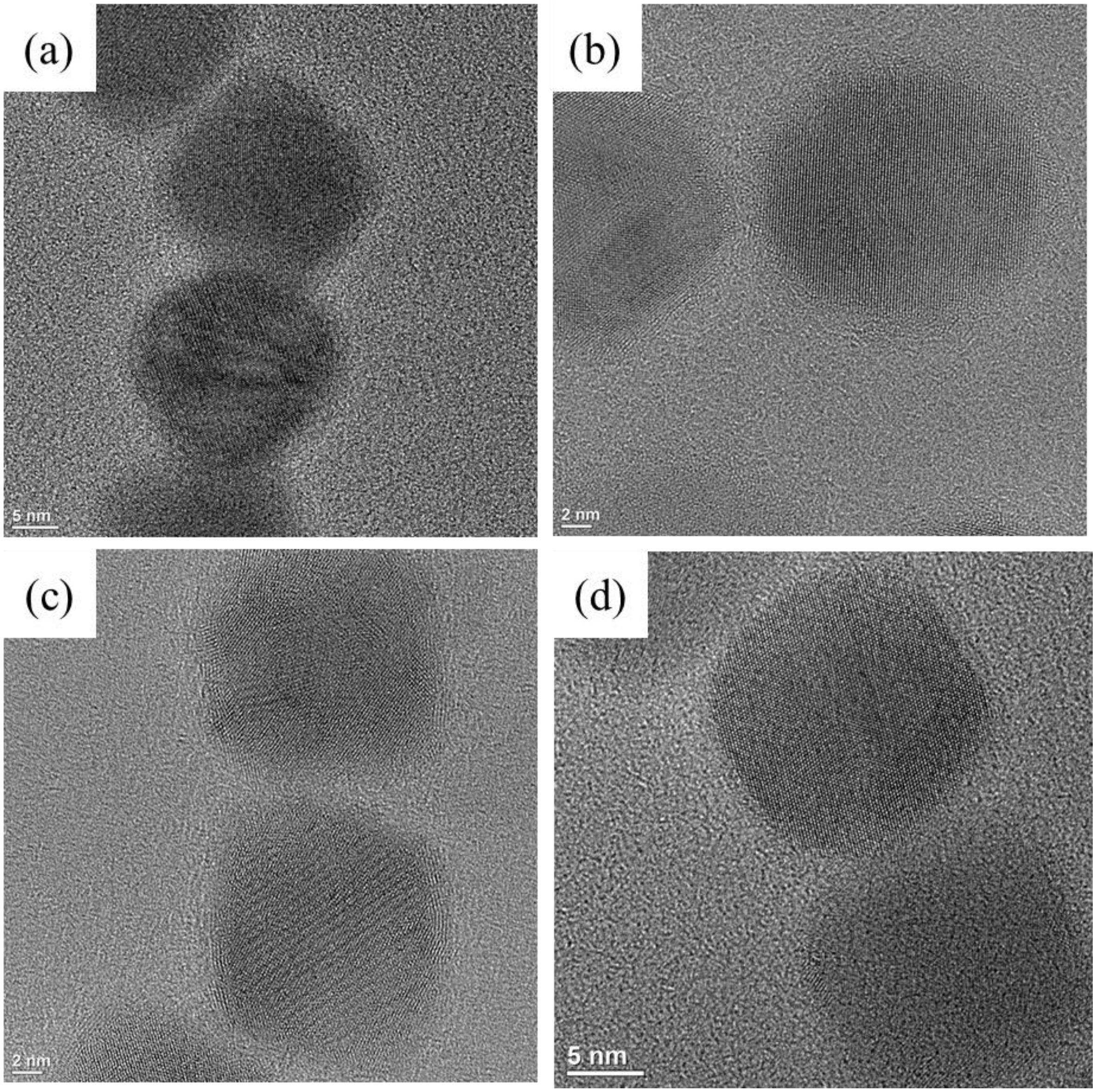

Figure S6. HR-TEM images of PS_{4500}-Co NPs: (a-c) NPs synthesized with the concentration of cobalt carbonyl fixed at 21.9 mM, and with different concentration of $PS\text{-}NH_2$ (Mn = 4500 g/mol, C_{PS}): (a) C_{PS} = 5 mM; (b) C_{PS} = 2.5 mM; (c) C_{PS} = 1.25 mM. (d) NPs synthesized with the concentration of $PS\text{-}NH_2$ at 2.5 mM, and cobalt carbonyl at 7.3 mM. The PS-Co NP samples were prepared on TEM grids from respective Co NP dispersion in DCB.

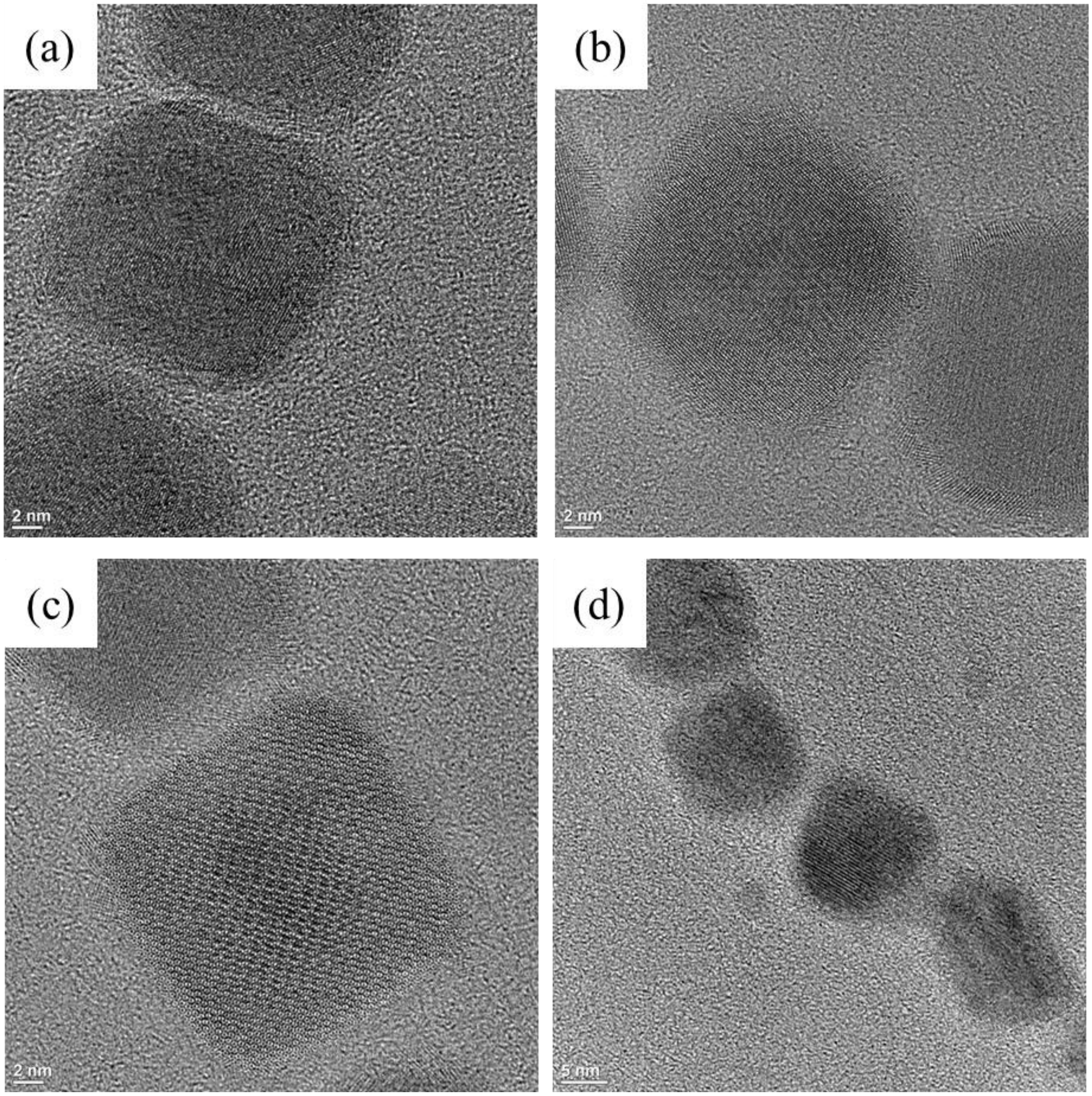

Figure S7. HR-TEM images of PS_{10500}-Co NPs synthesized with the concentration of cobalt carbonyl fixed at 21.9 mM, and with different concentration of $PS-NH_2$ (Mn = 10500 g/mol, C_{PS}): (a) C_{PS} = 5 mM; (b) C_{PS} = 2.5 mM; (c) C_{PS} = 1.25 mM and (d) C_{PS} = 0.67 mM The PS-Co NP samples were prepared on TEM grids from respective Co NP dispersion in DCB.
